# Development of Narcolepsy Type 1 Following COVID-19 Infection in a Patient Previously Diagnosed With Idiopathic Hypersomnia: A Case Report

**DOI:** 10.7759/cureus.113053

**Published:** 2026-07-20

**Authors:** Binh Pham, Talha Memon

**Affiliations:** 1 Sleep Medicine, Southwest Healthcare Medical Education Consortium, Temecula, USA

**Keywords:** autoimmune, cataplexy, covid-19, idiopathic hypersomnia (ih), narcolepsy type 1, pediatric obstructive sleep apnea, pediatric sleep disorders, sodium oxybate for narcolepsy

## Abstract

Narcolepsy type 1 is a rare sleep disorder characterized by excessive daytime sleepiness (EDS) and cataplexy, thought to result from the immune-mediated destruction of hypocretin-producing neurons in genetically susceptible individuals. Viral infections have been implicated as potential environmental triggers. We present the case of a 17-year-old female National Collegiate Athletic Association (NCAA) Division I student-athlete with a prior diagnosis of idiopathic hypersomnia who developed new-onset cataplexy following COVID-19 infection. Initial polysomnography and multiple sleep latency testing demonstrated severe hypersomnolence without sleep-onset rapid eye movement periods. Several months later, she developed emotionally triggered episodes of muscle weakness consistent with cataplexy. Repeat multiple sleep latency testing demonstrated only one sleep-onset rapid eye movement period; however, given the development of typical cataplexy and the overall clinical presentation, a clinical diagnosis of narcolepsy type 1 was made. Cerebrospinal fluid hypocretin testing was not performed. Symptoms were refractory to multiple stimulant and antidepressant regimens but responded well to sodium oxybate in combination with solriamfetol, resulting in the resolution of EDS and cataplexy. This case highlights a possible temporal association between COVID-19 infection and the subsequent development of narcolepsy type 1 and underscores the importance of clinical judgment when objective diagnostic testing is inconclusive.

## Introduction

Narcolepsy is a chronic neurologic disorder characterized by excessive daytime sleepiness (EDS) and, in narcolepsy type 1, cataplexy resulting from hypocretin deficiency. Medical literature suggests that narcolepsy has an autoimmune basis, particularly in genetically susceptible individuals carrying the HLA-DQB1*06:02 allele [1]. Several recent case reports have described new-onset narcolepsy following COVID-19 infection, suggesting that virus-induced immune dysregulation may contribute to disease onset; however, a definitive causal relationship has not been established [2]. Treatment approaches for narcolepsy and other hypersomnias are guided by established practice parameters, including the use of wake-promoting agents and sodium oxybate for the management of EDS and cataplexy [3]. Narcolepsy is classified according to established diagnostic criteria that distinguish between narcolepsy type 1 and type 2 based on clinical features and objective sleep testing [4]. Previous studies have associated infections such as *Streptococcus pyogenes *and influenza A (H1N1) with an increased incidence of narcolepsy, supporting the hypothesis that infections may trigger autoimmune responses affecting hypocretin-producing neurons in genetically susceptible individuals [1,5]. Although the exact mechanism remains under investigation, accumulating case reports describing narcolepsy following COVID-19 infection are consistent with this hypothesis but do not establish causality [1,2,5].

A preliminary version of this work was previously presented as a poster at the 39th Annual Meeting of the Associated Professional Sleep Societies (SLEEP 2025) and published as a conference abstract [6].

## Case presentation

A 17-year-old female National Collegiate Athletic Association (NCAA) Division I student-athlete, with a medical history of generalized anxiety disorder (GAD), allergic rhinitis, and a previous sinus surgery, presented with complaints of snoring and EDS. She initially sought evaluation at a sleep clinic and was referred to an ear, nose, and throat (ENT) clinic for symptoms indicative of allergic rhinitis. She later sought a second opinion at our facility due to persistent daytime sleepiness that had significantly impacted both her athletic and academic performance. The patient reported struggling with excessive sleepiness since the age of 12, but her symptoms had worsened recently.

Her sleep routine typically involved going to bed around 10:00 PM and waking up at 7:00 AM on weekdays (9:00 AM on weekends), with a sleep onset latency of approximately 20 minutes. She consistently took daily naps, lasting between one and four hours on school days and at least twice on weekends. Her Epworth Sleepiness Scale (ESS) score was 18/24.

The patient denied symptoms of cataplexy, hypnopompic hallucinations, hypnagogic hallucinations, sleep paralysis, or fragmented sleep. She reported occasional snoring but had no complaints of restless legs syndrome or parasomnias. There was no history of dream-enactment behaviors.

The patient underwent initial polysomnography (PSG) and multiple sleep latency testing (MSLT), which revealed idiopathic hypersomnia with a mean sleep latency of three minutes and 50 seconds, without instances of sleep-onset rapid eye movement (REM) periods. Her baseline PSG did not indicate sleep-disordered breathing.

Following the diagnosis of idiopathic hypersomnia, the patient was prescribed modafinil 200 mg daily. At the two-month follow-up visit, she reported significant improvements in functionality and a reduction in daytime sleepiness. Her school performance and athletic abilities had improved.

However, six months later, she returned with worsening symptoms of daytime sleepiness despite modafinil titration. She reported that the medication had become ineffective. Her school grades declined due to oversleeping and missing classes. Additionally, she experienced new-onset muscle weakness. She described episodes of "passing out" prior to cheerleading performances, during which her knees buckled, but she remained conscious. Similar episodes occurred when she was socializing, such as when her boyfriend told her a joke. She denied any bowel or bladder incontinence, memory loss, or confusion during these episodes. These spells occurred 3-4 times a week, each lasting approximately one minute, with no facial droop noted by witnesses.

Upon further inquiry, the patient reported a COVID-19 infection approximately three months prior. This additional history, together with her newly reported episodes suggestive of cataplexy, prompted further diagnostic evaluation with repeat PSG and MSLT. Details regarding the severity of the infection, hospitalization status, vaccination history, antiviral treatment, and duration of illness were not available in the medical record.

Physical exam

The patient was alert, oriented, well-nourished, and well-groomed, with no signs of somnolence during the evaluation. Vital signs included the following: blood pressure 111/73 mmHg, pulse 60 bpm, and BMI 19.5 kg/m². The head, eyes, ears, nose, and throat (HEENT) exam showed a normal nasopharyngeal cavity with no evidence of congestion, a Mallampati class I oropharynx, and 1+ tonsils bilaterally. The rest of the physical exam was unremarkable.

The differential diagnosis included narcolepsy type 2; however, the subsequent development of typical cataplexy favored narcolepsy type 1. Epileptic seizures, psychogenic nonepileptic seizures, and functional neurologic disorder were also considered. However, the episodes were consistently triggered by strong emotions, occurred without loss of consciousness, postictal confusion, or other features suggestive of seizures, making these diagnoses less likely. Syncope was considered but was not supported by the absence of transient loss of consciousness or cardiovascular symptoms. Medication-induced symptoms were also considered, but the timing of symptom onset following COVID-19 infection and persistence despite multiple medication adjustments made this less likely. Insufficient sleep syndrome was considered; however, the patient reported adequate habitual sleep duration with extended weekend sleep and persistent hypersomnolence despite sufficient opportunity for sleep. Circadian rhythm sleep-wake disorder was also considered, but the patient maintained a relatively consistent sleep schedule without significant phase delay or advance, making this diagnosis less consistent with her presentation.

Additional diagnostic studies, including cerebrospinal fluid (CSF) hypocretin measurement, HLA-DQB1*06:02 testing, brain magnetic resonance imaging (MRI), and electroencephalogram (EEG), were considered but not performed. Although these investigations may provide additional diagnostic support in selected cases, the patient's characteristic clinical presentation, particularly the development of emotionally triggered cataplexy, was considered sufficient to support a clinical diagnosis of narcolepsy type 1. We acknowledge that the absence of CSF hypocretin testing and human leukocyte antigen (HLA) typing limits diagnostic certainty and represents a limitation of this report. Should the patient's clinical course change or diagnostic uncertainty persist, these additional investigations may be considered in the future.

## Discussion

Cataplexy, observed in nearly 87% of patients, is often triggered by laughter (as in this case), with excitement (65%) and stress (54%) also being common triggers [5]. Muscle weakness typically affects the legs and knees, but in children, symptoms may include perioral and tongue movements, facial grimaces, ptosis, and tongue protrusion [5].

Narcolepsy diagnosis can be challenging, with an average delay of 10 years from symptom onset to diagnosis [5]. The impact on daily functioning, including school and work, can be severe. Although our patient did not meet the classic International Classification of Sleep Disorders (ICSD)-3 diagnostic criteria for narcolepsy type 1 based on MSLT findings alone, the subsequent development of typical emotionally triggered cataplexy, persistent EDS, and the emergence of a sleep-onset REM period on repeat MSLT supported the clinical diagnosis [4]. We acknowledge that the absence of CSF hypocretin testing limits diagnostic certainty and represents a limitation of this report.

Prior to COVID-19 infection, PSG showed good sleep efficiency with preserved sleep architecture and no evidence of clinically significant sleep-disordered breathing or periodic limb movements (Table 1).

**Table 1:** Pre-COVID-19 diagnostic polysomnography findings SE: sleep efficiency; TST: total sleep time; N1-N3: sleep stages; REM: rapid eye movement; AHI: apnea-hypopnea index; NREM: non-rapid eye movement; RDI: respiratory disturbance index; PLMI: periodic limb movement index Normal adult values include sleep efficiency >85%, AHI <5 events/hour, and PLMI <15 events/hour. This study demonstrated normal sleep efficiency, preserved sleep architecture, and no evidence of clinically significant sleep-disordered breathing or periodic limb movement disorder.

Polysomnography findings	
SE (%)	97.3
TST (min)	429.4
N1 (%)	3.4
N2 (%)	43.8
N3 (%)	30.3
REM (%)	22.6
REM latency (min)	47.2
AHI	3.1
RDI	9.9
Supine AHI/nonsupine AHI	N/A
NREM AHI/REM AHI	3.4/1.9
Oxygen nadir	92%
PLMI	2.4

Despite these normal nocturnal findings, MSLT demonstrated severe hypersomnolence with a mean sleep latency of three minutes and 50 seconds and no sleep-onset REM periods, supporting an initial diagnosis of idiopathic hypersomnia (Table 2).

**Table 2:** Pre-COVID-19 multiple sleep latency test SOREMP: sleep-onset rapid eye movement period; REM: rapid eye movement Normal mean sleep latency is >8 minutes. The patient's mean sleep latency was markedly reduced (3 minutes and 50 seconds) without the presence of SOREMP, consistent with severe hypersomnolence and supporting an initial diagnosis of idiopathic hypersomnia.

Nap	Sleep latency (min:sec)	SOREMP	REM latency (min:sec)
1	2:48	No	N/A
2	1:48	No	N/A
3	4:18	No	N/A
4	0:54	No	N/A
5	9:24	No	N/A
Mean	3:50	0/5	N/A

After the patient developed COVID-19, repeat PSG again showed no obstructive sleep apnea, though REM sleep was reduced and REM latency was markedly prolonged (Table 3).

**Table 3:** Post-COVID-19 diagnostic polysomnography findings SE: sleep efficiency; TST: total sleep time; N1-N3: sleep stages; REM: rapid eye movement; AHI: apnea-hypopnea index; NREM: non-rapid eye movement; RDI: respiratory disturbance index; PLMI: periodic limb movement index Normal adult values include sleep efficiency >85%, AHI <5 events/hour, and PLMI <15 events/hour. Repeat polysomnography demonstrated preserved sleep efficiency and no clinically significant obstructive sleep apnea or periodic limb movements, although REM sleep percentage was reduced (13.5%) and REM latency was markedly prolonged (223.5 minutes).

Polysomnography findings	
SE (%)	87.5
TST (min)	450.5
N1 (%)	4.3
N2 (%)	58.3
N3 (%)	23.9
REM (%)	13.5
REM latency (min)	223.5
AHI	2.8
RDI	13.7
Supine AHI/nonsupine AHI	3.0/1.5
NREM AHI/REM AHI	2.5/0.0
Oxygen nadir	94%
PLMI	0

Follow-up MSLT continued to show pathologically short sleep latency with the emergence of a sleep-onset REM period, supporting progression from idiopathic hypersomnia to a clinical diagnosis of narcolepsy type 1 (Table 4).

**Table 4:** Post-COVID-19 multiple sleep latency test SOREMP: sleep-onset rapid eye movement period; REM: rapid eye movement; ICSD: International Classification of Sleep Disorders Normal mean sleep latency is >8 minutes. Follow-up multiple sleep latency test demonstrated persistently reduced mean sleep latency (5 minutes and 57 seconds) with the emergence of one SOREMP. Although the patient did not fulfill the classic ICSD-3 multiple sleep latency test criteria for narcolepsy type 1, these findings, together with the development of typical cataplexy, supported the clinical diagnosis.

Nap	Sleep latency (min:sec)	SOREMP	REM latency (min:sec)
1	7:18	No	N/A
2	4:48	No	N/A
3	7:00	Yes	14:30
4	4:36	No	N/A
5	6:06	No	N/A
Mean	5:57	1/5	14:30

The patient was initially treated with escitalopram for cataplexy, and her modafinil dosage was adjusted. However, other regimens, including methylphenidate and Adderall, did not effectively control her symptoms. Eventually, sodium oxybate was introduced, leading to significant improvements in daytime sleepiness, cataplexy, and overall functionality [3]. Subsequent follow-up visits included a trial of automatic positive airway pressure (APAP) (5-10 cm H₂O) to address mild sleep-disordered breathing that could have contributed to her residual symptoms. The patient reported an approximately 10% subjective improvement in overall sleep quality and remained compliant with therapy; however, APAP therapy alone did not adequately control her EDS or cataplexy.

Narcolepsy with cataplexy (narcolepsy type 1) is a rare disorder, affecting approximately 0.02% of the population globally [5]. Despite its rarity, the disability associated with this condition can be significant, with up to 24% of affected individuals experiencing severe impairment [5]. Cataplexy is a hallmark feature of narcolepsy type 1 and is often triggered by emotional stimuli, such as laughter [5]. This differentiates it from narcolepsy type 2 and idiopathic hypersomnia.

The pathophysiology of narcolepsy with cataplexy is thought to involve the destruction of hypothalamic neurons producing hypocretin-1 and hypocretin-2, neuroendocrine hormones that regulate sleep-wake cycles, appetite, and energy balance [1,5]. Our patient's longstanding history of EDS, without cataplexy, initially led to a diagnosis of idiopathic hypersomnia. However, after COVID-19 infection, she developed cataplectic episodes, raising the possibility of a temporal association between the infection and the onset of narcolepsy. Recent reports by Wang et al. and Künstler et al. have similarly described patients who developed narcolepsy following COVID-19 infection, although these observations do not establish a causal relationship [7,8]. One proposed mechanism is immune dysregulation affecting hypocretin-producing neurons in genetically susceptible individuals, although a causal relationship remains unproven [1,2].

Recent literature suggests a potential autoimmune basis for hypocretin cell loss in narcolepsy. In particular, narcolepsy has been associated with the HLA-DQB1*06:02 allele and may be triggered by infections, such as *Streptococcus pyogenes *or influenza [1,5]. The COVID-19 infection could have induced a proinflammatory response that led to the overactivation of cytotoxic T cells, which may have attacked the hypothalamic neurons producing hypocretin [1,2].

Narcolepsy post-COVID-19 infection has been reported globally, with several cases showing similar symptom onset following viral infections [2]. Our patient was successfully treated with sodium oxybate, an FDA-approved treatment for both EDS and cataplexy [3]. After titrating the dose to 9 g nightly, combined with daytime solriamfetol 150 mg, the patient experienced complete resolution of her daytime sleepiness and cataplexy [3].

## Conclusions

The combination of sodium oxybate (9 g nightly) and solriamfetol (150 mg daily) was associated with marked improvement in this patient's daytime sleepiness and cataplexy, allowing her to resume an active lifestyle. This case highlights a possible temporal association between COVID-19 infection and the subsequent development of narcolepsy type 1, although a causal relationship cannot be established. While sodium oxybate and solriamfetol were effective in this patient, additional studies are needed to better understand treatment outcomes in similar cases. Despite not meeting the classic American Academy of Sleep Medicine (AASM) diagnostic criteria for narcolepsy type 1, the patient's clinical presentation, particularly the development of typical cataplexy, supported the diagnosis. Continued research into the relationship between viral infections, immune dysregulation, and narcolepsy is needed to further improve diagnosis and management.
